# Predictive modelling of laser powder bed fusion of Fe-based nanocrystalline alloys based on experimental data using multiple linear regression analysis

**DOI:** 10.1016/j.heliyon.2024.e35047

**Published:** 2024-07-22

**Authors:** Merve G. Özden, Xianyuan Liu, Tom J. Wilkinson, Meryem S. Üstün-Yavuz, Nicola A. Morley

**Affiliations:** a Department of Material Science and Engineering, University of Sheffield, Sheffield, S1 3JD, UK; b Department of Computer Science, University of Sheffield, Sheffield, S1 4DP, UK; c Centre of Machine Intelligence, University of Sheffield, Sheffield, S1 4DA, UK; d School of Allied Health Professions, Nursing & Midwifery, University of Sheffield, Sheffield, S10 2TS, UK

**Keywords:** Design of experiment, Bivariate correlational analysis, Multiple linear regression analysis, Laser powder bed fusion and Fe-based nano-crystalline materials

## Abstract

This study harnessed bivariate correlational analysis, multiple linear regression analysis and tree-based regression analysis to examine the relationship between laser process parameters and the final material properties (bulk density, saturation magnetization (*M*_*s*_), and coercivity (*H*_*c*_)) of Fe-based nano-crystalline alloys fabricated via laser powder bed fusion (LPBF). A dataset comprising of 162 experimental data points served as the foundation for the investigation. Each data point encompassed five independent variables: laser power (*P*), laser scan speed (*v*), hatch spacing *(h*), layer thickness (*t*), and energy density (*E*), along with three dependent variables: bulk density, *M*_*s*_, and *H*_*c*_. The bivariate correlational analysis unveiled that bulk density exhibited a significant correlation with *P*, *v*, *h*, and *E*, whereas *M*_*s*_ and *H*_*c*_ displayed significant correlations exclusively with *v* and *P*, respectively. This divergence may stem from the strong influence of microstructure on magnetic properties, which can be impacted not only by the laser process parameters explored in this study but also by other factors such as oxygen levels within the build chamber. Furthermore, our statistical analysis revealed that bulk density increased with rising *P*, *h*, and *E*, while decreased with higher *v*. Regarding the magnetic properties, a high *M*_*s*_ was achievable through low *v*, while low *H*_*c*_ resulted from high *P*. It was concluded that *P* and *v* were considered as the primary laser process parameters, influencing *h* and *t* due to their control over the melt-pool size. The application of multiple linear regression analysis allowed the prediction of the bulk density by using both laser process parameters and energy density. This approach offered a valuable alternative to time-consuming and costly trial-and-error experiments, yielding a low error of less than 1 % between the mean predicted and experimental values. Although a slightly higher error of approximately 6 % was observed for *M*_*s*_, a clear association was established between *M*_*s*_ and *v*, with lower *v* values corresponding to higher *M*_*s*_ values. Additionally, a further comparison was conducted between multiple linear regression and three tree-based regression models to explore the effectiveness of these approaches.

## 1 Introduction

Laser powder bed fusion (LPBF), also referred to as selective laser melting (SLM), stands as the cornerstone within the realm of metal additive manufacturing (metal-AM). This primacy owes itself to its remarkable capability to build intricate shapes while maintaining mechanical properties that meet industry standards. LPBF distinguishes itself from other metal-AM techniques by being the preeminent choice across diverse sectors including automotive, energy, medical, and aviation [[1], [2], [3]].

**Fig. 1:**
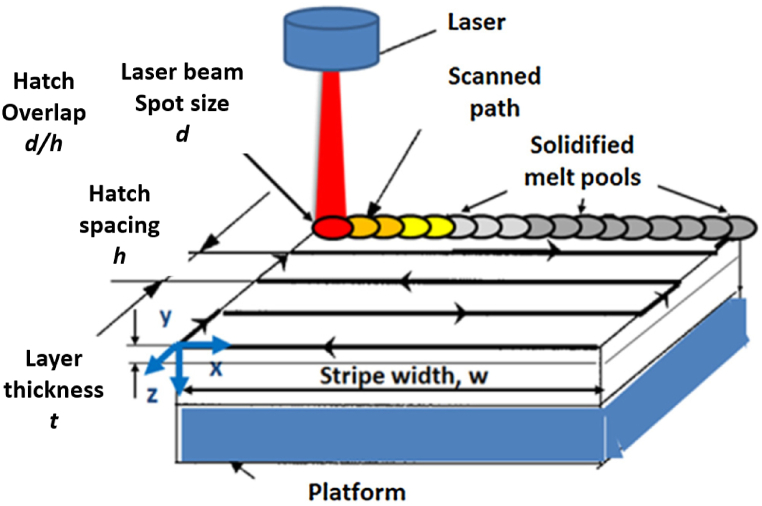
The schematic illustrations of the LPBF process showing the major build parameters [9].

In the LPBF procedure, a slender layer of metal powder is evenly distributed onto a building platform using a spreading tool. Subsequently, a laser beam is employed to scan across this layer, harnessing its thermal energy to meticulously liquefy selected portions of the powdered material. This sequential process is replicated layer by layer, gradually shaping the desired 3D geometry as the laser beam liquefies and merges successive layers (Fig. 1) [[4], [5], [6]].

The physical transformations that occur as the metal powders transition into a consolidated form encompasses several stages, including laser absorption, melting, vaporization, solidification, and re-heating/melting. Precise control of these physical processes necessitates the fine adjustment of machine process parameters to achieve the desired quality of the LPBF component [7].

Certainly, optimizing the LPBF process parameters is a complex endeavour that necessitates extensive trial and error experiments. The efficacy of process optimization in LPBF is contingent upon several factors, including the type of machinery employed, the characteristics of the powder material, and the specific build geometry being pursued. It's important to note that this optimization process must be revisited whenever any of the variables undergoes a change.

Moreover, it has been observed that more than 130 distinct process parameters exert a significant influence on the LPBF process. Among these, the major build parameters such as laser power (*P*), laser scan speed (*v*), beam spot size (*d*), hatch spacing (*h*), and layer thickness (*t*) are well-recognized for their impact on aspects like melt pool geometry, the mode of melting, vaporization, and the formation of physical defects [8].

Regarding the range of values for these parameters employed in LPBF, *P* typically varies between 50 and 400 W, *v* spans the range of 100–2500 mm/s, and *d* often falls within the 50–100 μm range. The choice of *h* is constrained by the diameters of the laser beam spot and the width of the melt pool, while layer thickness (*t*) is contingent upon factors such as powder size and the depth of the melt pool. In addition, a volumetric energy input, also known as energy density (*E*), equation (Eqn (1)) has been utilized to combine all the major process parameters.

(1) $$E=\frac{P}{vht}$$

Soft-magnetic materials play a crucial role in electronic products and exert a significant impact on human life [10,11]. While silicon steel dominates the soft magnetic materials market owing to its low-cost, its effective permeability (*μ*_*e*_) remains insufficient, resulting in significant power generation and transmission losses. In contrast, amorphous alloys exhibit high permeability and minimal loss, making them an ideal substitute for silicon steel [12]. Among amorphous alloys, Fe-based alloys offer higher saturation magnetic flux density than FeNi-based and Co-based ones. Consequently, this competitive benefit positions Fe-based amorphous alloys as suitable candidates for applications in distribution transformers, intermediate frequency transformers, pulse transformers, and filter inductors [13,14].

In order to lower the low-frequency losses observed in Fe-based amorphous alloys and enhance their soft magnetic behaviour, researchers have introduced Fe-based nanocrystalline alloys. In 1988, Yashizawa pioneered the development of a Fe-based nanocrystalline alloy through heat treatment of Fe-based amorphous alloys [15]. The structure of Fe-based nanocrystalline alloys consists of an amorphous phase combined with an α-Fe crystalline phase [16]. When the exchange interaction length exceeds the grain size of nanocrystalline alloys, their effective anisotropy constant becomes extremely small, reducing coercivity (*H*_*c*_). Meanwhile, the permeability of the alloy becomes inversely proportional to its coercivity [17].

Based on the theory of magnetism, the soft-magnetic materials exhibit favourable stress sensitivity and maintain high magnetic permeability when their magnetostriction coefficient (*λ*_*s*_) approaches zero. The distinctive structure of nanocrystalline alloys allows for the cancellation of positive magnetostriction in the amorphous phase and negative magnetostriction in the nanocrystalline phase. This intriguing property holds the promise of producing soft magnetic materials with zero magnetostriction and significantly improved overall soft magnetic characteristics [18]. Compared to Fe-based amorphous alloys, Fe-based nanocrystalline alloys offer advantages such as higher magnetic induction, greater permeability, and lower coercivity [11].

These materials find applications in low-frequency, high-power magnetic devices like transformers and switching power supplies, and are also well-suited for giant magnetoimpedance sensing devices. As a result, the advent of Fe-based nanocrystalline alloys marked a substantial breakthrough in the evolution of soft magnetic alloys, driving the development of amorphous alloys to new heights [19]. As a recent focus of research, the production of Fe-based nanocrystalline alloys without dimensional limitations and the need of time-consuming, several step-containing and costly processing is being studied. Laser additive manufacturing can overcome all these constraints. However, its laser process parameters including laser power and scan speed need to be optimized in order to obtain high bulk density as well as superior soft-magnetic properties (high saturation magnetization (*M*_*s*_) and low coercivity (*H*_*c*_)).

In order to ascertain the most optimum processing parameters, the creation of additive manufacturing (AM) models becomes imperative. Chen [20] has classified AM modelling investigations into three distinct categories: empirical, analytical, and numerical models, as well as incorporating machine learning methodologies. Empirical models, particularly when dealing with materials like metal powders [21], can incur substantial costs when conducting practical experiments. This elevated cost factor contributes to the increased complexity of identifying the parameters that exert an impact on the quality of the final manufactured components [22]. Several attempts have been made to predict density [[23], [24], [25], [26]], surface quality [7,27,28], mechanical properties [[29], [30], [31], [32], [33]] and melt-pool size and geometry [9,[34], [35], [36]] using statistical tools such as design of experiments (DoE). This process can be enhanced through the utilization of machine learning (ML). ML models, employing various algorithms, can be employed to unearth patterns within data and utilize this acquired knowledge to make predictions regarding specific values based on data that hasn't been previously observed. Within the realm of supervised learning, regression models are constructed to predict continuous variables as responses, while classification models are exclusively tailored for categorical variables [37,38]. Among these, the regression model offers a straightforward and readily interpretable output analysis, although it relies on the assumption of a linear relationship between the parameters. Linear regression primarily examines the connection between the mean of outcome variables (final material properties) and independent variables (laser process parameters) [30]. Also, its error and predictability values are comparable to ML techniques [25]. For this reason, this study utilizes statistical tools including multiple linear regression analysis to investigate the relationship between the laser process parameters and final material properties (magnetic properties and bulk density) of LPBF-processed Fe-based nanocrystalline alloys based on the experimental data. To provide a comprehensive analysis, the study compares the performance of multiple linear regression with that of tree-based regression [39,40], adding further discussion to explore the effectiveness of each approach in detail.

## 2 Experimental procedure

A total of 162 experimental data points were generated using different combinations of the five process parameters. These data points were obtained from the three published papers [[41], [42], [43]]. The alloy composition is fixed as KUAMET 6B2 (Fe_87.38_Si_6.85_B_2.54_Cr_2.46_C_0.77_ (mass %)). Across the experimental data set the laser power (*P*) ranged between 30 and 150 W, laser scan speed (*v*) ranged between 500 and 1300 mm/s, hatch spacing (*h*) ranged between 0.02 and 0.06 mm, layer thickness (*t*) ranged between 0.03 and 0.07 mm and energy density (*E*) ranged between and 31.81 and 103.17 J/mm^3^. The complete list of process parameters employed during the LPBF processing can be found in Appendix A.

A bivariate correlational analysis was carried out to investigate the relationships between the five process parameters and the three outcome variables. This was followed by a multiple linear regression and tree-based analysis where the potential influence of these five process parameters on the final properties of Fe-based nanocrystalline alloys, particularly on bulk density, saturation magnetization and coercivity, was investigated. Since energy density is calculated using laser power, laser speed, hatch spacing and layer thickness and is not an independent parameter (at least not to degree of the other four parameters), it was determined to run two different multiple linear regressions; one using the four independent process parameters and another one only using the energy density.

## 3 Results

### 3.1 Relationship between the process parameters and the outcome variables

The magnitude of the Pearson correlation coefficient (*r*) determines the strength of the correlation. To assess the strength of association some general guidelines have been provided by Cohen [44] which suggest that if 0.1 < |*r* | < 0.3 the strength of association is small, if 0.3 < |*r* | < 0.5 the strength of association is medium and if |*r* | > 0.5 the strength of association is strong. Table 1 shows the relationship between the five process parameters and the three material properties. A significant small correlation has been observed between bulk density and four of the process parameters; laser power (*r* = 0.22), laser scan speed (*r* = 0.14), hatch spacing (*r* = 0.16) and energy density (*r* = 0.18). The relationship between the bulk density and laser power, hatch speed and energy density was a positive one meaning that when one of these process parameters increased, the bulk density also increased whereas the relationship between bulk density and laser scan speed was a negative one meaning that when laser scan speed increased, the bulk density decreased. When it comes to the relationship between the five process parameters and saturation magnetization a significant small correlation was observed only between the saturation magnetization and laser scan speed (*r* = 0.24). This relationship was a negative one meaning that when laser scan speed increased, the saturation magnetization (*M*_*s*_) decreased and vice versa. Finally, a significant small correlation was observed between the coercivity (*H*_*c*_) and laser power (*r* = 0.14). This relationship was negative in nature meaning that when laser power increased, the coercivity decreased and vice versa. There was not a significant correlation between the remaining four process parameters and coercivity.

**Table 1:** Relationship between the process parameters and outcome variables.

Pearson's Correlations (r)			
	Bulk density (%)	*M*_*s*_ (Am^2^/kg)	*H*_*c*_ (kA/m)
Laser power (W)	0.221^b^	−0.057	−0.138^a^
Laser scan speed (mm/s)	−0.141^a^	−0.237^b^	−0.117
Hatch spacing (mm)	0.163^a^	−0.015	0.063
Layer thickness (mm)	−0.070	0.077	−0.127
Energy density (J/mm^3^)	0.179^a^	0.042	−0.045

Note.

a *p* ≤ .05.

b *p* ≤ .01 (one -tailed).

### 3.2 Prediction modelling

#### 3.2.1 Prediction modelling using process parameters

Multiple regression analyses were carried out to verify and further investigate the predictive relationships between the four independent variables (laser speed (*P*), laser scan speed (*v*), hatch spacing (*h*), layer thickness (*t*)) and the three outcome variables (bulk density, saturation magnetization, coercivity). The four process parameters statistically significantly predicted the bulk density and saturation magnetization but not the coercivity. Hence, only bulk density and saturation magnetization will be discussed in more detail.

The model for bulk density was highly significant, *F*(4,157) = 6.685, *p* < 0.001, the process parameters explaining 15 % of the overall variance in bulk density. Significant predictors in the order of their relative contributions are laser power (*β* = 0.477), laser scan speed (*β* = −0.344) and layer thickness (*β* = −0.281) (Table 2). Hatch spacing was not a unique predictor of bulk density. Since the unstandardised coefficient (*B)* represents the change in the dependent variable for a one unit change in the independent variable a regression equation predicting the bulk density based on the different values of the four process parameters can be formulated as follows.

(2) **Mean bulk density (%)** = 99.286 + (.021 x *P*) + (−.002 x *v*) + (−14.862 x *h*) + (−26.306 x *t*)

**Table 2:** Multiple linear regression - Bulk density and the four process parameters.

Predictor	*Unstandardised Coefficient* (*B)*	*Standardised Coefficient* (*β)*	p value	95.0 % Confidence Interval for B	
				Lower Bound	Upper Bound
**(Model) *R***^***2***^ = 0***.146***			<0.001		
**(Constant)**	99.286		<0.001	97.628	100.943
**Laser power (W)**	0.021	0.477	<0.001	0.012	0.031
**Laser scan speed (mm/s)**	−0.002	−0.344	<0.001	−0.003	−0.001
**Hatch spacing (mm)**	−14.862	−0.144	0.181	−36.682	6.957
**Layer thickness (mm)**	−26.306	−0.281	0.008	−45.489	−7.123

**Table 3:** Multiple linear regression - Saturation magnetization and the four process parameters.

Predictor	*Unstandardised Coefficient* (*B*)	*Standardised Coefficient* (*β*)	p value	95.0 % Confidence Interval for (B)	
				Lower Bound	Upper Bound
(Model) *R*^*2*^ = 0*.061*			0.041		
(Constant)	190.199		<0.001	168.565	211.833
Laser power (W)	0.015	0.027	0.814	−0.113	0.144
Laser scan speed (mm/s)	−0.018	−0.246	0.009	−0.032	−0.005
Hatch spacing (mm)	−17.157	−0.013	0.905	−301.956	267.643
Layer thickness (mm)	65.318	0.056	0.607	−185.071	315.707

**Table 4:** Simple linear regression - Bulk density and energy density.

Predictor	*Unstandardised Coefficient* (*B*)	*Standardised Coefficient* (*β*)	p value	95.0 % Confidence Interval for (B)	
				Lower Bound	Upper Bound
(Model) *R*^*2*^ = 0*.032*					
**(Constant)**	96.771		<0.001	96.067	97.474
**Energy density (J/mm**^**3**^**)**	0.013	0.179	0.023	0.002	0.024

The above equation can be used to calculate the predicted value of bulk density, which can also be referred to as the expected bulk density and it is the predicted mean bulk density. However, there can be other factors such as laser spot size, gas flow rate, chamber pressure, scan length and direction impacting the bulk density, which in turn can result in some variation. The range of the predicted variation in the mean bulk density can be calculated based on the confidence intervals. This means that 95 % confidence can be expressed in the accuracy of the actual mean of bulk density, as determined by the four process parameters, being situated within the lower (minimum value) and upper bounds (maximum value), which can be calculated as below.

(3) **Min. bulk density (%)** = 97.628 + (.012 x *P*) + (−.003 x *v*) + (−36.682 x *h*) + (−45.489 x *t*)

(4) **Max. bulk density (%)** = 100.943 + (.031 x *P*) + (−.001 x *v*) + (6.957 x *h*) + (−7.123 x *t*)

The model for saturation magnetization was significant, *F*(4,157) = 2.563, *p* = 0.041, the process parameters explaining 6 % of the overall variance in saturation magnetization. The only significant predictor was laser scan speed (*β* = −0.246). The other three process parameters were not an unique predictor of saturation magnetization. Since the unstandardised coefficient (*B)* represents the change in the dependent variable for a one unit change in the independent variable, a regression equation predicting the magnetization saturation based on the different values of the four process parameters can be formulated.

(5) **Mean *M***_***s***_**(Am**^**2**^**/kg)** = 190.199 + (.015 x *P*) + (−.018 x *v*) + (−17.157 x *h*) + (65.318 x *t*)

Equation (5) can be employed for computing the anticipated saturation magnetization value, also known as the expected mean saturation magnetization. Like bulk density, there may be additional factors that influence saturation magnetization (*M*_*s*_), leading to some degree of variability. By utilizing confidence intervals, the potential range of variation in the mean saturation magnetization can be estimated. This implies that 95 % confidence in the accuracy of the true mean saturation magnetization can be expressed based on the four process parameters, falling within the lower (min.) and upper (max.) bounds, as demonstrated below.

(6) **Min. *M***_***s***_**(Am**^**2**^**/kg)** = 168.565 + (−.113 x *P*) + (−.032 x *v*) + (−301.956 x *h*) + (−185.071 x *t*)

(7) **Max. *M***_***s***_**(Am**^**2**^**/kg)** = 211.833 + (.144 x *P*) + (−.005 x *v*) + (267.643 x *h*) + (315.707 x *t*)

#### 3.2.2 Prediction modelling using energy density

Since energy density is one single parameter, instead of multiple linear regression analysis, simple linear regression was conducted to assess the predictability of energy density over the three outcome variables. The regression analysis showed that energy density only statistically significantly predicted the bulk density. Hence only this will be discussed in further detail.

Energy density (*E*) statistically significantly predicted bulk density, *F*(4,160) = 5.287, *p* = 0.023, and explained 3 % of the overall variance in the bulk density. Given that the unstandardised coefficient (*B*) signifies how the dependent variable changes with a one-unit alteration in the independent variable, it is possible to create a regression equation that predicts bulk density by considering the variation in energy density.

(8) **Mean bulk density (%)** = 96.771 + (.013 x *E*)

Similarly, there can be additional factors that can influence energy density and its prediction power of bulk density leading to some variability. The predicted range of variation in the mean bulk density can be quantified using confidence intervals with the upper and lower bounds, as outlined below.

(9) **Min. bulk density (%)** = 96.067 + (.002 x *E*)

(10) **Max. bulk density (%)** = 97.474 + (.024 x *E*)

**Fig. 2:**
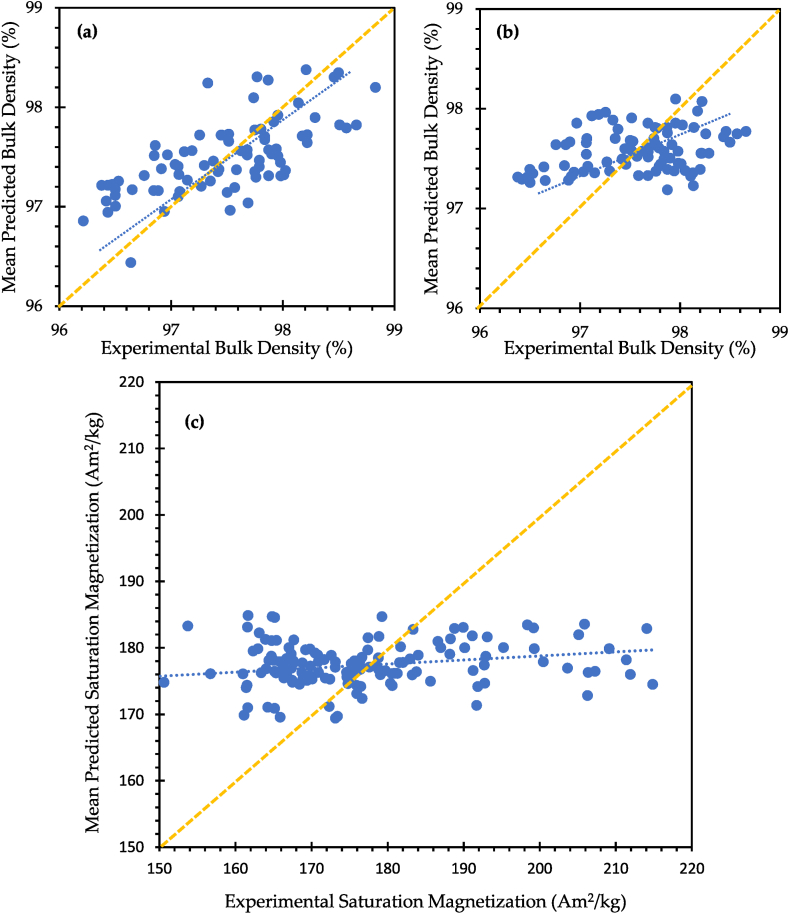
The graphs of the mean predicted bulk density versus the experimental bulk density; the mean predicted bulk density calculated using **(a)** equation (2) and **(b)** equation (8); and **(c)** the graph showing the relationship between experimental saturation magnetization and mean predicted saturation magnetization quantified using equation (5).

Fig. 2 illustrates the mean predicted bulk density values quantified using equation (2) (Fig. 2(a)) and equation (8) (Fig. 2(b)) as a function of the experimental bulk density. Furthermore, Fig. 2(c) demonstrates the relationship between experimental *M*_*s*_ and mean predicted *M*_*s*_ calculated using equation (5).

### 3.3 Comparative analysis of different regression methods

Several tree-based methods were carried out to compare the effectiveness of the multiple linear regression, including decision tree regression, random forest regression, and XGBoost regression [39,40]. The results are shown in Table 5.

**Table 5:** Comparison with tree-based methods using mean squared error (MSE) and mean absolute error (MAE). The best result for each row is in **bold**, and the second best is underlined. The results are the average outcomes obtained using different random seeds, ranging from 0 to 99.

Methods		Decision Tree Regression [39]	Random Forest Regression [39]	XGBoost Regression [40]	Multiple Linear Regression
***E* → *B***	**MSE**	2.059	1.612	1.812	**1.292**
	**MAE**	1.079	0.968	1.001	**0.875**
***Pvht* → *B***	**MSE**	1.266	**0.820**	0.841	1.187
	**MAE**	0.712	0.621	**0.584**	0.817
***Pvht* → *M***_***s***_	**MSE**	307.668	**193.697**	261.418	212.599
	**MAE**	12.142	**10.687**	11.316	11.449

Multiple linear regression achieves the best performance among tree-based methods on the bulk density (*B*) prediction using a single variable, i.e. energy density (*E*).

Tree-based methods, especially random forest regression, demonstrated enhanced capabilities in handling multi-variable (*Pvht*) scenarios, such as laser power (*P*), laser scan speed (*v*), hatch spacing (*h*), and layer thickness (*t*). However, multiple linear regression still outperforms decision tree regression across most metrics and achieves comparable results to XGBoost in predicting saturation magnetization, showing its effectiveness in narrowing the search space while providing straightforward outputs.

### 3.4 SHAP analysis

SHAP (SHapley Additive exPlanations) analysis was used to interpret the contributions of individual features to the output of machine learning models [45]. Fig. 4 shows the SHAP analysis of each prediction conducted in the previous section. The SHAP values are plotted on the horizontal axis, which measures the impact of each feature, while the vertical axis labels the features being analysed. The colour scale, from blue to red, indicates the feature values, with blue representing lower and red representing higher values.

**Fig. 3:**
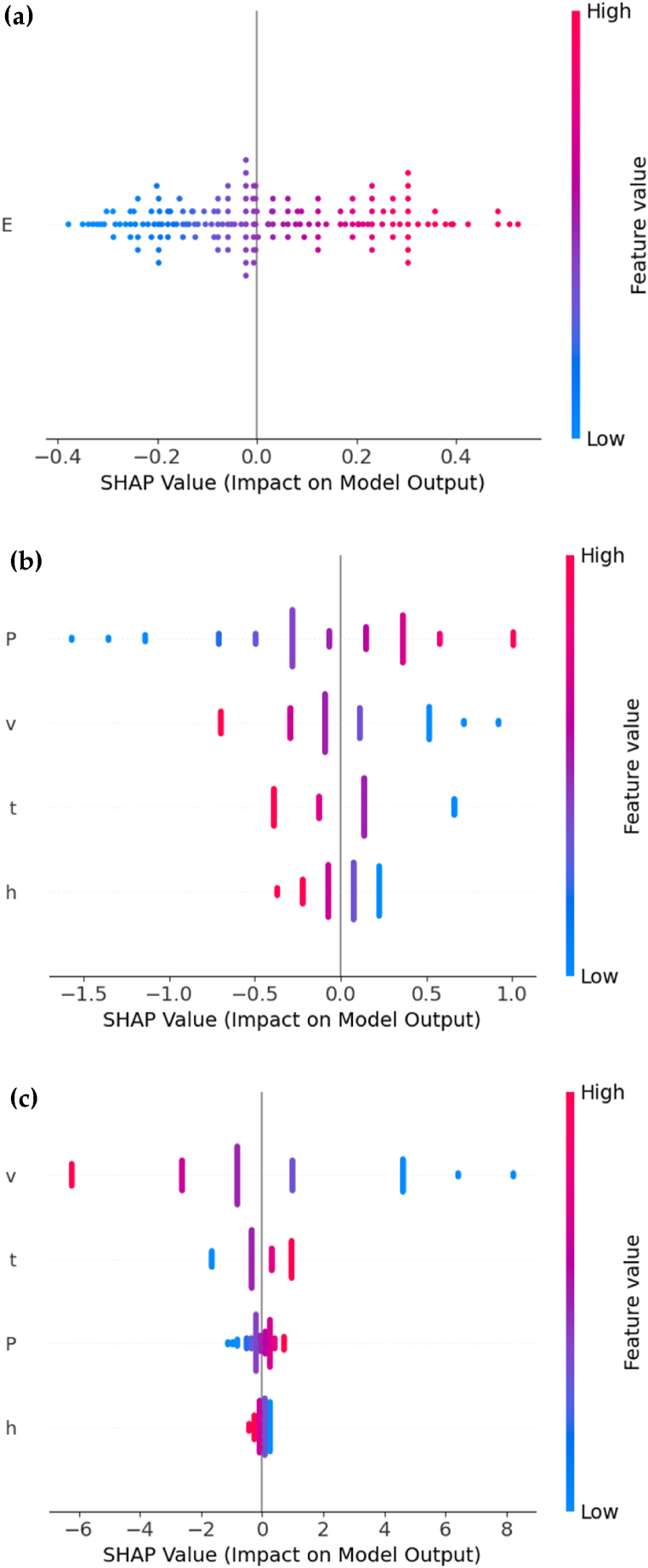
SHAP value distribution plots of different tasks for Multiple Linear Regression, showing the importance of different variables: **(a)** Using energy density (*E*) for prediction of bulk density (*B*), **(b)** using laser power (*P*), laser scan speed (*v*), hatch spacing (*h*), and layer thickness (*t*) for prediction of bulk density (*B*) and **(c)** using laser power (*P*), laser scan speed (*v*), hatch spacing (*h*), and layer thickness (*t*) for prediction of saturation magnetization (*M*_*s*_).

As shown in Fig. 3(a), the values range from approximately −0.4 to 0.4 and an increase in energy density *E* positively impacts the model output. However, the overlap in data points suggests non-linear interactions or other feature interdependencies might influence the relationship. In Fig. 3(b), laser power (*P*) positively affects the output, while laser scan speed (*v*), hatch spacing (*h*), and layer thickness (*t*) negatively affect it. The data points for *P* show a symmetrical spread around zero, indicating a balanced influence on the predictions. In contrast, the data points for *v*, *t*, and *h* are more concentrated around zero, showing a moderate but less pronounced impact compared to *P*. This result aligns with Equation (1). For predicting saturation magnetization, as shown in Fig. 3(c), laser scan speed (*v*) has a high negative impact, with widespread data points indicating a strong influence on the predictions. Layer thickness (*t*) positively impacts the output, with other factors having minimal influence.

**Fig. 4:**
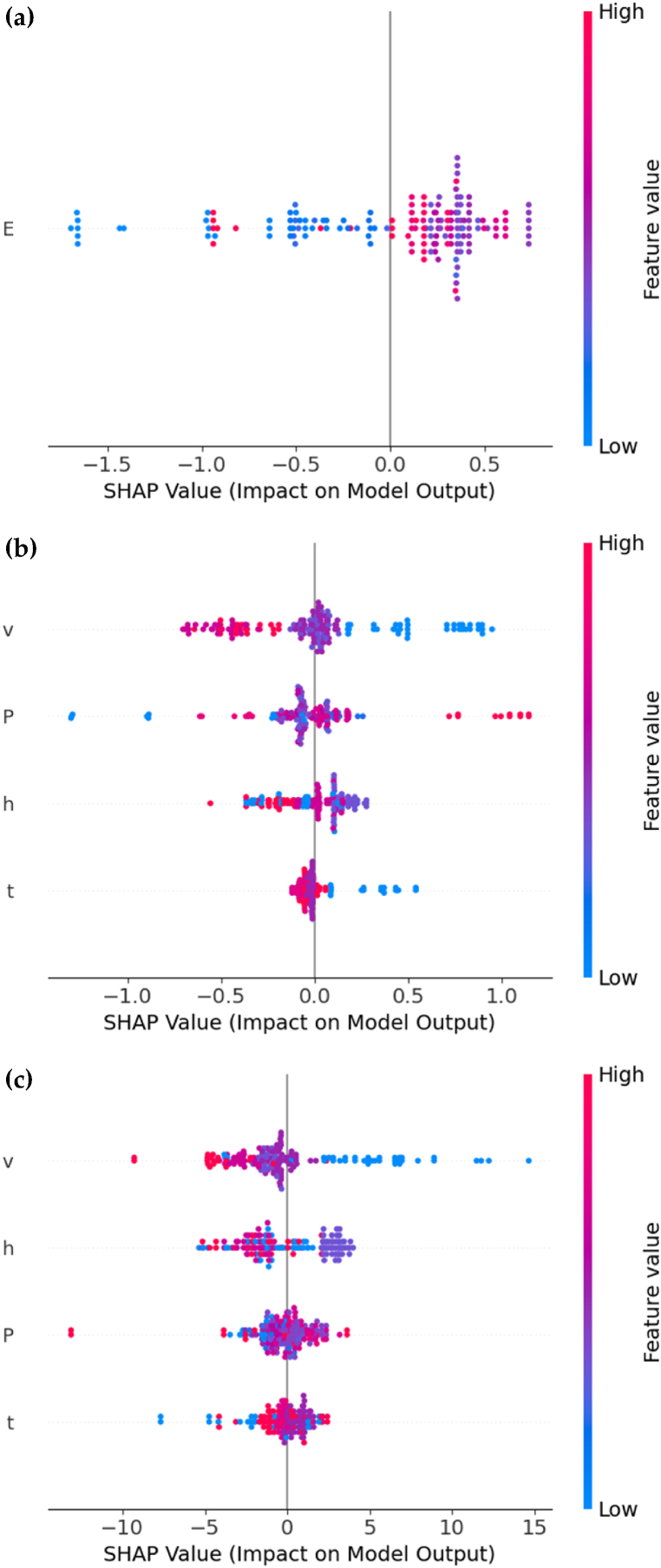
SHAP value distribution plots of different tasks for Random Forest Regression, showing the importance of different variables: **(a)** Using energy density (*E*) for prediction of bulk density (*B*), **(b)** using laser power (*P*), laser scan speed (*v*), hatch spacing (*h*), and layer thickness (*t*) for prediction of bulk density (*B*) and **(c)** using laser power (*P*), laser scan speed (*v*), hatch spacing (*h*), and layer thickness (*t*) for prediction of saturation magnetization (*M*_*s*_).

Fig. 4 presents the SHAP analysis of Random Forest Regression, showing the non-linear relationship. As shown in Fig. 4(a), lower energy density (*E*) results in a lower output, while moderate to high values lead to a higher output. In Fig. 4(b), laser power (*P*), laser scan speed (*v*), and layer thickness (*t*) exhibit similar trends to those in Fig. 3(b), while hatch spacing (*h*) displays some mixed clustering. Fig. 4(c) indicates that laser scan speed (*v*) still has a high negative impact, whereas other factors have minimal influences. The positive factor of layer thickness (*t*) shows minimal influence in non-linear relationships.

Comparing Fig. 3, Fig. 4, we found that most variables exhibit similar trends, highlighting the effectiveness of Multiple Linear Regression, but there are inconsistencies in certain variables. These inconsistencies are primarily due to the nonlinear relationships and interactions present in the data. Random Forest Regression captures these nonlinear relationships and complex interactions between variables, whereas Multiple Linear Regression assumes linear relationships.

## 4 Discussion

This study successfully performed the design of experiments (DoE) of final properties of LPBF-processed Fe-based nanocrystalline alloys with 162 data points. Firstly, the Pearson's correlation allowed to define the relationship between the major laser process parameters and the final properties. It was found that the bulk density significantly depends on all the process parameters, except layer thickness (*t*). The fact that high bulk density can be obtained with increasing laser power and (*P*) and decreasing laser scan speed (*v*) complies with the findings in literature [41,42]. However, experimentally the high hatch spacing (*h*) worsened the bulk density when other process parameters kept constant [41]. The reason for the statistical result of increasing bulk density with increasing *h* can be its dependence on other process parameters, especially *P*, even though, theoretically they are independent from each other. It was suggested that the optimal *h* value is determined based on the melt-pool size, which is influenced mostly by *P* and slightly by *v* [42]. Similar to *h*, *t* is also affected by *P* and *v*. This may be the reason why there was no statistically significant relationship between *t* and the final properties. Furthermore, a significant negative correlation between saturation magnetization (*M*_*s*_) and *v* was expected as high *v* causes high cooling rate, which in turn increases amorphous content, reducing *M*_*s*_ [42]. On the other hand, it was anticipated a significant positive relationship between coercivity (*H*_*c*_) and *P* instead of a negative relationship. Since the magnetic properties (*M*_*s*_ and *H*_*c*_) substantially depend on the microstructure including the impurities, porosities and the presence of the phases, it may be a good idea to consider other process conditions such as the oxygen level of the chamber and the oxygen content in the parent powder in order to comprehend the change in the magnetic properties. Although this work proposed that statistically *M*_*s*_ is only influenced by *v* and *H*_*c*_ is only affected by *P*, the effect of the other laser process parameters cannot be ruled out. All process conditions must be taken into consideration together with *P*, *v*, *h* and *t* as a whole to examine the change in the microstructure and so, magnetic behaviour. Energy density is also taken into account as an independent variable. It only had a significant correlation with bulk density. The positive correlation supported the experimental results [41].

The predictability analysis was initially conducted using multiple linear regression analysis. According to this analysis, only hatch spacing was not a predictor for bulk density. Laser power has both the most and only positive contributions on bulk density, meaning that *P* possesses the most impact on the predicted bulk density and increasing *P* increases bulk density. Both *v* and *t* have a negative contribution to the predicted bulk density. These results do not contradict the literature. Moreover, as expected from the Pearson's correlation, energy density predicted only the bulk density with positive contribution.

The error between the mean predicted bulk density based on the laser process parameters and the experimental one was calculated as 0.84 ± 0.75 %, whereas the error of 0.9 ± 0.78 % was found when quantifying the predicted bulk density based on energy density (*E*). Although this implies that the bulk density can be predicted accurately across the reported range by utilizing equation (2) or equation (8), the author acknowledges that 162 data points used occupy a narrow search space in terms of bulk density range (≈2 %) and that the associated errors over this range are therefore not insignificant. This may be due to the relationships being non-linear over a more comprehensive power/bulk density range when factors such as the formation of lack of fusion pores (low power) and keyhole pored (high power) are considered [46]. While it is possible that the relationship over the analysed range is not entirely linear, Fig. 2(a) appears to show that these external factors do not significantly affect the bulk density range of the data used in this study.

Fig. 2(b) shows the use of energy density to predict bulk density. This yields far inferior results to laser processing parameters. However, this is not surprising, as energy density has previously been reported as an unreliable parameter for material production by the laser powder bed fusion technique [47], a statement that this study supports.

The prediction analysis was also carried out for saturation magnetization based on the laser process parameters. As the laser scan speed was the only predictor for *M*_*s*_, the error between the mean predicted *M*_*s*_ values and the experimental values is higher (6.16 ± 4.81 %) than that of bulk density. As illustrated in Fig. 2(c), saturation magnetization prediction based solely on laser process parameters (*P*, *v*, *h* and *t*) is not quite accurate as its deviation from the experimental saturation magnetization is rather high. Other parameters like powder characteristics and microstructural properties might need to be considered for multiple linear regression analysis. On the other hand, it is obvious that the laser scan speed (*v*) has a negative contribution to the predicted *M*_*s*_, same as the negative Pearson's correlation between *v* and *M*_*s*_. This indicates that statistically, *v* has a definite impact on *M*_*s*_ and higher *v* brings about lower *M*_*s*_ when the other laser process parameters are constant.

As mentioned earlier in this section, soft-magnetic behaviour strongly depends on the microstructural properties such as porosity levels, amorphous phase content and crystallite size. Including these data into prediction modelling may produce more accurate results in predicting soft-magnetic properties of LPBF-processed Fe-based nanocrystalline alloys. This is because LPBF process parameters influence the microstructural development [48]. There are several evidence that decreasing laser power and increasing laser scan speed enhances amorphous phase content in the microstructure [42,49,50]. Furthermore, relatively larger hatch spacing and layer thickness increases the amorphous phase fraction [42]. In other words, low energy density (*E*) promotes vitrification [50], decreasing coercivity (*H*_*c*_) [49]. On the other hand, high amorphous phase content (i.e., low *E*) reduces saturation magnetization (*M*_*s*_) [42]. Nanocrystallization is a solution to maximize *M*_*s*_ without increasing *H*_*c*_. Increasing *P* and decreasing *v* increases crystallite size and lowers amorphous phase content, which improves *M*_*s*_, but worsens *H*_*c*_. Keeping the mean crystallite size below 200 nm lowers coercivity to 130 A/m, where *M*_*s*_ is around 165 Am^2^/kg [43]. Alternatively, high amorphous phase content (89.6 %) can result in low coercivity (397 A/m) [51]. As a result, soft-magnetic behaviour, especially coercivity, is quite difficult to predict based only on process parameters. This is likely due to the well-known dependence of coercivity on the evolution of the microstructure [52].

To assess the overall accuracy of the multiple regression model, the coefficient of determination (*R*^*2*^) values in Table 2, Table 3, Table 4 can be examined. *R*^*2*^ indicates the proportion of variance in the outcome variable (bulk density or *M*_*s*_) that can be predicted based on the values of the predictor variables (*P*, *v*, *h*, *t* and *E*). For bulk density prediction, *R*^*2*^ values are 0.146 and 0.032 in Table 2, Table 4, respectively. This means that with the laser process parameters (*P*, *v*, *h*, *t*) predictor variables, 14.6 % of the variance can be predicted, while only 3.2 % of the variance can be predicted with the energy density predictor variables in the measure of bulk density. The model based on laser process parameters explains a larger portion of the variance in the bulk density. In the case of saturation magnetization (*M*_*s*_) prediction, *R*^*2*^ value is 0.061 in Table 3, implying that 6.1 % of the variance can be predicted in the measure of *M*_*s*_ by using the multiple linear regression model based on process parameters.

Furthermore, the comparison between multiple linear regression and tree-based regression models, as presented in Table 5, underscores their differing prediction capabilities and shows that multiple linear regression achieves performance comparable to other methods. Additionally, SHAP analysis based on multiple linear regression, as shown in Fig. 3, has a similar observation as Pearson's correlation analysis, further demonstrating its reliability in providing interpretable results. Therefore, due to its ease of implementation and computational efficiency, multiple linear regression is an ideal choice in scenarios that require rapid model development and deployment, or when computational resources are limited. Its simplicity also facilitates more straightforward assessments of statistical significance and confidence intervals for predictor variables. This is essential for understanding the robustness of the predictive factors and for communicating results in an interpretable manner. Therefore, although tree-based methods may excel in some complex scenarios, multiple linear regression remains a valuable tool for its clarity and practicality.

## 5 Conclusion

This research employed the bivariate correlational analysis and the multiple linear regression analysis and tree-based methods to explore the correlation between laser process parameters and the final material characteristics (bulk density, saturation magnetization (*M*_*s*_) and coercivity (*H*_*c*_)) of Fe-based nanocrystalline alloys produced through LPBF, using 162 experimental data points as the basis for investigation. Each experimental data point contained 5 independent variables; laser power (*P*), laser scan speed (*v*), hatch spacing (*h*), layer thickness (*t*) and energy density (*E*); and 3 dependent variables; bulk density, *M*_*s*_ and *H*_*c*_. The bivariate correlational analysis showed that while bulk density has a significant correlation with *P*, *v*, *h* and *E*; *M*_*s*_ and *H*_*c*_ significantly correlate only with *v* and *P*, respectively. This may be because magnetic properties excessively depend on the microstructure and microstructural evolution is affected by not only the laser process parameters studied in this paper but also other process conditions like the oxygen level in the building chamber. Moreover, statistically bulk density increases with increasing *P*, *h* and *E*; and decreasing *v*. In the case of magnetic properties, high *M*_*s*_ can be achieved by low *v* and low *H*_*c*_ resulted from high *P*. It was concluded that *P* and *v* are the main laser process parameters, on which *h* and *t* depend owing to *P* and *v* controlling the melt-pool size. With the help of multiple linear regression analysis, the predicted bulk density can be obtained utilizing laser process parameters, replacing the time-consuming and high-cost trial and error experiments due to the low error (<1 %) between the mean predicted and experimental values. Despite the higher error for M_s_ was higher (6.16 %), a clear link was observed between *M*_*s*_ and *v*; the lower *v* the higher *M*_*s*_. Further exploration with tree-based models and SHAP analysis further verified the effectiveness of multiple linear regression and provided guidance for future experiments. These findings show that machine learning was successfully implemented to enhance initial processing parameter selection in LPBF optimization.

## CRediT authorship contribution statement

**Merve G. Özden:** Writing – review & editing, Writing – original draft, Visualization, Validation, Resources, Project administration, Methodology, Funding acquisition, Formal analysis, Data curation, Conceptualization. **Xianyuan Liu:** Writing – review & editing, Visualization, Validation, Supervision, Software, Methodology, Investigation, Formal analysis, Data curation, Conceptualization. **Tom J. Wilkinson:** Writing – review & editing, Visualization, Validation, Methodology, Investigation, Formal analysis, Data curation, Conceptualization. **Meryem S. Üstün-Yavuz:** Writing – original draft, Visualization, Validation, Methodology, Investigation, Formal analysis, Data curation, Conceptualization. **Nicola A. Morley:** Writing – review & editing, Supervision, Resources, Project administration, Methodology, Conceptualization.

## Declaration of competing interest

The authors declare the following financial interests/personal relationships which may be considered as potential competing interests:Merve Gizem Ozden reports financial support was provided by Republic of Turkey Ministry of National Education. Merve Gizem Ozden reports was provided by The University of Sheffield. If there are other authors, they declare that they have no known competing financial interests or personal relationships that could have appeared to influence the work reported in this paper.

## Figures and Tables

**Table:** 

Sample No	*P* (W)	*v* (mm/s)	*h* (mm)	*t* (mm)	*E* (J/mm^3^)	Bulk Density (%)	*M*_*s*_ (Am^2^/kg)	*H*_*c*_ (kA/m)
1	90	1000	0.02	0.05	90	98.94	207.27	1.82
2	90	1300	0.02	0.05	69.23076923	97.53	164.25	2.39
3	90	700	0.03	0.05	85.71428571	99	178.86	1.88
4	90	1000	0.03	0.05	60	98.51	206.38	2.15
5	90	1300	0.03	0.05	46.15384615	95.64	165.15	1.78
6	120	1000	0.03	0.05	80	98.14	176.41	1.57
7	120	1300	0.03	0.05	61.53846154	97.98	191.71	1.16
8	90	700	0.04	0.05	64.28571429	98.99	177.42	1.65
9	90	1000	0.04	0.05	45	98.13	180.57	2.30
10	120	700	0.04	0.05	85.71428571	99.25	205.13	2.46
11	120	1000	0.04	0.05	60	98.29	191.25	2.60
12	120	1300	0.04	0.05	46.15384615	97.76	172.33	1.92
13	90	700	0.05	0.05	51.42857143	97.46	188.27	1.92
14	120	700	0.05	0.05	68.57142857	98.5	191.14	2.27
15	120	1000	0.05	0.05	48	95.7	179.24	2.07
16	120	1300	0.05	0.05	36.92307692	92.4	161.61	1.50
17	150	700	0.05	0.05	85.71428571	96.61	163.12	1.81
18	150	1000	0.05	0.05	60	98.21	164.03	1.71
19	90	700	0.06	0.05	42.85714286	97.68	167.68	1.62
20	120	700	0.06	0.05	57.14285714	98.83	193.11	2.33
21	120	1300	0.04	0.05	46.15384615	97.76	172.33	1.92
22	90	1300	0.03	0.05	46.15384615	95.64	165.15	1.78
23	120	700	0.06	0.05	57.14285714	98.83	193.11	2.33
24	120	1000	0.03	0.07	57.14285714	98.96	175.65	1.54
25	150	1000	0.05	0.05	60	98.21	164.03	1.71
26	90	1000	0.05	0.03	60	98.22	180.38	2.11
27	90	1000	0.03	0.05	60	98.51	206.38	2.15
28	120	1000	0.04	0.05	60	98.29	191.25	2.60
29	90	700	0.03	0.07	61.2244898	98.7	199.2	1.89
30	120	700	0.04	0.07	61.2244898	98.93	153.74	1.81
31	150	700	0.05	0.07	61.2244898	99.44	205.89	1.88
32	120	1300	0.03	0.05	61.53846154	97.98	191.71	1.16
33	120	1300	0.05	0.03	61.53846154	97.84	173.41	1.74
34	90	700	0.04	0.05	64.28571429	98.99	177.42	1.65
35	90	1000	0.02	0.07	64.28571429	97.69	167.69	1.40
36	90	700	0.06	0.03	71.42857143	97.74	199.28	1.75
37	150	1300	0.03	0.07	54.94505495	99.38	175.94	1.75
38	120	1300	0.04	0.03	76.92307692	98.66	161.14	2.14
39	150	1300	0.05	0.03	76.92307692	98.46	139.2	2.30
40	120	1000	0.05	0.03	80	97.87	169.4	1.91
41	120	1000	0.03	0.05	80	98.14	176.41	1.57
42	90	700	0.05	0.03	85.71428571	97.33	195.24	2.76
43	90	700	0.03	0.05	85.71428571	99	178.86	1.88
44	120	700	0.04	0.05	85.71428571	99.25	205.13	2.46
45	90	1000	0.04	0.03	75	98.57	167.49	1.77
46	90	1300	0.04	0.03	57.69230769	97.57	173.14	1.41
47	100	1300	0.04	0.03	64.1025641	97.79	165.87	1.72
48	120	1300	0.04	0.03	76.92307692	98.66	161.14	2.14
49	90	700	0.05	0.03	85.71428571	97.33	195.24	2.76
50	90	1000	0.05	0.03	60	98.22	180.38	2.11
51	100	1000	0.05	0.03	66.66666667	97.92	150.61	1.80
52	120	1000	0.05	0.03	80	97.87	169.4	1.91
53	120	1300	0.05	0.03	61.53846154	97.84	173.41	1.74
54	150	1300	0.05	0.03	76.92307692	98.46	139.2	2.30
55	90	700	0.06	0.03	71.42857143	97.74	199.28	1.75
56	100	700	0.06	0.03	79.36507937	97.77	167.04	1.94
57	100	1000	0.06	0.03	55.55555556	97.84	174.8	1.74
58	90	1000	0.02	0.07	64.28571429	97.69	167.69	1.40
59	90	1300	0.02	0.07	49.45054945	96.64	176.65	1.31
60	120	1300	0.02	0.07	65.93406593	99.27	206.29	1.59
61	90	700	0.03	0.07	61.2244898	98.7	199.2	1.89
62	120	700	0.03	0.07	81.63265306	99.23	198.38	1.65
63	120	1000	0.03	0.07	57.14285714	98.96	175.65	1.54
64	150	1000	0.03	0.07	71.42857143	99.32	178.79	1.79
65	150	1300	0.03	0.07	54.94505495	99.38	175.94	1.75
66	120	700	0.04	0.07	61.2244898	98.93	153.74	1.81
67	150	1000	0.04	0.07	53.57142857	99.13	182.95	1.94
68	120	700	0.05	0.07	48.97959184	98.51	161.58	2.05
69	150	700	0.05	0.07	61.2244898	99.44	205.89	1.88
70	70	900	0.02	0.05	77.77777778	99.38	176.35	1.75
71	70	1000	0.02	0.05	70	97.50	180.88	2.60
72	70	1100	0.02	0.05	63.63636364	96.43	161.53	2.38
73	80	900	0.02	0.05	88.88888889	97.12	164.39	1.87
74	80	1000	0.02	0.05	80	97.75	174.6	1.91
75	80	1100	0.02	0.05	72.72727273	98.44	168.42	1.87
76	90	900	0.02	0.05	100	96.23	167.36	1.96
77	90	1000	0.02	0.05	90	97.19	165.13	1.70
78	90	1100	0.02	0.05	81.81818182	98.02	192.77	1.67
79	70	900	0.03	0.05	51.85185185	98.61	182.02	1.35
80	70	1000	0.03	0.05	46.66666667	98.05	211.91	2.39
81	70	1100	0.03	0.05	42.42424242	98.11	176.48	1.47
82	80	900	0.03	0.05	59.25925926	97.06	170.9	1.65
83	80	1000	0.03	0.05	53.33333333	97.27	181.27	2.07
84	80	1100	0.03	0.05	48.48484848	98.50	175.93	2.34
85	90	900	0.03	0.05	66.66666667	96.86	165.06	2.14
86	90	1000	0.03	0.05	60	96.17	170.92	1.82
87	90	1100	0.03	0.05	54.54545455	96.43	214.85	2.44
88	70	900	0.04	0.05	38.88888889	98.41	175.21	1.90
89	70	1000	0.04	0.05	35	98.13	169.78	2.10
90	70	1100	0.04	0.05	31.81818182	97.87	161.41	2.54
91	80	900	0.04	0.05	44.44444444	96.53	181.57	1.71
92	80	1000	0.04	0.05	40	96.42	179.06	1.88
93	80	1100	0.04	0.05	36.36363636	96.21	191.86	2.07
94	90	900	0.04	0.05	50	97.79	165.83	1.55
95	90	1000	0.04	0.05	45	97.15	166.73	2.20
96	90	1100	0.04	0.05	40.90909091	95.40	180.61	2.47
97	90	900	0.02	0.06	83.33333333	99.15	170.81	1.58
98	90	1000	0.02	0.06	75	98.26	166.27	1.79
99	90	1100	0.02	0.06	68.18181818	97.06	168.87	1.73
100	100	900	0.02	0.06	92.59259259	98.18	167.33	1.99
101	100	1000	0.02	0.06	83.33333333	97.96	178.47	1.78
102	100	1100	0.02	0.06	75.75757576	97.87	166.37	1.63
103	110	900	0.02	0.06	101.8518519	97.96	170.47	1.96
104	110	1000	0.02	0.06	91.66666667	97.26	192.71	1.64
105	110	1100	0.02	0.06	83.33333333	96.97	174.65	1.70
106	90	900	0.03	0.06	55.55555556	97.42	164.72	1.67
107	90	1000	0.03	0.06	50	97.08	203.66	1.55
108	90	1100	0.03	0.06	45.45454545	96.94	175.19	2.39
109	100	900	0.03	0.06	61.72839506	97.63	184.03	1.55
110	100	1000	0.03	0.06	55.55555556	97.42	177.6	1.80
111	100	1100	0.03	0.06	50.50505051	96.84	170.09	1.90
112	110	900	0.03	0.06	67.90123457	97.75	188.18	1.85
113	110	1000	0.03	0.06	61.11111111	97.68	169.86	1.81
114	110	1100	0.03	0.06	55.55555556	96.24	171.82	1.61
115	90	900	0.04	0.06	41.66666667	98.35	176.62	1.87
116	90	1000	0.04	0.06	37.5	96.50	178.81	2.72
117	90	1100	0.04	0.06	34.09090909	94.28	185.63	2.24
118	100	900	0.04	0.06	46.2962963	97.30	192.92	1.79
119	100	1000	0.04	0.06	41.66666667	96.38	167.35	2.66
120	100	1100	0.04	0.06	37.87878788	96.08	167.44	1.75
121	110	900	0.04	0.06	50.92592593	96.49	172.6	1.75
122	110	1000	0.04	0.06	45.83333333	97.04	173.12	1.92
123	110	1100	0.04	0.06	41.66666667	96.47	172.41	2.24
124	110	900	0.02	0.07	87.3015873	97.52	209.13	2.00
125	110	1000	0.02	0.07	78.57142857	97.38	167.09	1.75
126	110	1100	0.02	0.07	71.42857143	97.35	168.74	2.53
127	120	900	0.02	0.07	95.23809524	96.06	186.99	1.84
128	120	1000	0.02	0.07	85.71428571	95.05	171.65	2.17
129	120	1100	0.02	0.07	77.92207792	94.62	175.56	1.98
130	130	900	0.02	0.07	103.1746032	94.65	181.7	2.56
131	130	1000	0.02	0.07	92.85714286	95.25	166.71	2.40
132	130	1100	0.02	0.07	84.41558442	95.75	179.71	1.92
133	110	900	0.03	0.07	58.2010582	96.16	169.21	1.91
134	110	1000	0.03	0.07	52.38095238	97.98	200.47	1.90
135	110	1100	0.03	0.07	47.61904762	98.20	156.72	2.43
136	120	900	0.03	0.07	63.49206349	97.45	162.95	1.63
137	120	1000	0.03	0.07	57.14285714	97.68	167.6	1.87
138	120	1100	0.03	0.07	51.94805195	98.01	163.38	1.96
139	130	900	0.03	0.07	68.78306878	96.90	190.14	1.80
140	130	1000	0.03	0.07	61.9047619	97.52	211.39	1.92
141	130	1100	0.03	0.07	56.27705628	97.90	183.79	1.72
142	110	900	0.04	0.07	43.65079365	98.38	162.32	1.72
143	110	1000	0.04	0.07	39.28571429	96.89	168.8	1.89
144	110	1100	0.04	0.07	35.71428571	95.61	183.24	1.73
145	120	900	0.04	0.07	47.61904762	97.87	177.4	1.98
146	120	1000	0.04	0.07	42.85714286	97.59	173.14	2.07
147	120	1100	0.04	0.07	38.96103896	96.65	160.99	1.87
148	130	900	0.04	0.07	51.58730159	97.81	169.79	1.81
149	130	1000	0.04	0.07	46.42857143	97.94	167.32	1.82
150	130	1100	0.04	0.07	42.20779221	96.91	165.33	1.75
151	50	500	0.02	0.05	100	98.22	161.66	1.76
152	50	600	0.02	0.05	83.33333333	97.68	189.96	2.01
153	50	700	0.02	0.05	71.42857143	97.07	163.94	2.02
154	40	500	0.02	0.05	80	96.85	164.82	1.75
155	40	600	0.02	0.05	66.66666667	96.76	188.81	1.59
156	40	700	0.02	0.05	57.14285714	96.5	165.39	1.82
157	30	500	0.02	0.05	60	96.36	165.16	2.14
158	30	600	0.02	0.05	50	95.51	183.35	1.79
159	30	700	0.02	0.05	42.85714286	94.89	186.61	5.14
160	50	500	0.03	0.05	66.66666667	98.63	179.26	2.01
161	50	600	0.03	0.05	55.55555556	97.43	214.08	1.47
162	50	700	0.03	0.05	47.61904762	96.5	164.76	1.99

## Appendix A

The experimental dataset that was exploited in this research.
